# Preparation, Purification and Performance Evaluation of Polyclonal Antibody Against SARS-CoV-2 Produced in Rat

**DOI:** 10.34172/apb.2023.059

**Published:** 2022-11-04

**Authors:** Fatemeh Yaghoobizadeh, Mohammad Roayaei Ardakani, Mohammad Mehdi Ranjbar, Mohammad Khosravi, Hamid Galehdari

**Affiliations:** ^1^Department of Biology, Faculty of Sciences, Shahid Chamran University of Ahvaz, Ahvaz, Khouzestan, Iran.; ^2^Razi Vaccine and Serum Research Institute, Karaj, Alborz, Iran.; ^3^Department of Pathobiology, Faculty of Veterinary Medicine, Shahid Chamran University of Ahvaz, Ahvaz, Khouzestan, Iran.

**Keywords:** ELISA, SARS-CoV-2, Polyethylene glycol, Western blotting, Sucrose cushion, Virus precipitation

## Abstract

**Purpose:**

Among all known human coronaviruses, some viruses (e.g., SARS-CoV-2) cause severe pneumonia or even death. With the regard to its spread and the importance of its rapid identification/treatment, and because pAbs are relatively cheap, able to bind to more sites on antigens and even neutralize them, this study was done for the production and purification of anti-SARS-CoV-2 polyclonal antibodies (pAb) in rats.

**Methods:**

Viral antigen purification was performed by PEG/NaCl precipitation. The efficiency of the sucrose cushion method was also investigated to produce a purer antigen. Immunization was done and antibody purification was performed by ammonium sulfate precipitation (33%), dialysis, and ion-exchange chromatography. Western blotting and enzyme-linked immunosorbent assay (ELISA) were performed to verify the antibody specificity. All data were analyzed by SPSS software.

**Results:**

The results showed that the amount of concentrated virus increased with the increase of PEG concentration. Moreover, the sucrose cushion method increased its purity. Besides, induction of immune response in rats for pAb production with high titers was reached via these antigens and ELISA/western blot results indicated a suitable antibody-antigen interaction. Additionally, it was shown that ion-exchange chromatography could be a suitable technique for IgG purification.

**Conclusion:**

Herein, we presented a simple and cheap method for the purification of whole viral particles with relatively high quality. The results verified that these antigens could elicit a good immune response in the rat. The obtained pAbs showed a good specificity toward SARS-CoV-2 antigens. Accordingly, this study proposes a promising method for viral vaccine development.

## Introduction

 Among all known coronaviruses (CoVs), seven cases have the ability for human infection so far.^1-3^ Unlike four human CoVs (i.e. HCoV-OC43, HCoV-NL63, HCoV-229E, and HCoV-HKU1), MERS-CoV, SARS-CoV, and 2019-nCoV cause severe pneumonia or other organs’ failure and even death among the infected populations.^2,4^ The novel coronavirus (as a causative agent of pneumonia), which was introduced as 2019 coronavirus disease (COVID-19 or 2019-nCoV) by the World Health Organization (WHO) has been rapidly distributed on the epidemic scale since its first emergence in Wuhan, China and, it has been affected over 247 968 227 million people (http://covid19.who.int/) by 5 November 2021.^1,5,6^ The International Committee on Taxonomy of Viruses (ICTV), nominated the novel coronavirus as severe acute respiratory syndrome coronavirus 2 (SARS-CoV-2). On the 31^st^ of January 2020, WHO listed COVID-19 as PHEIC (Public Health Emergency of International Concern); It means that this virus may cause a threat to various countries, therefore combat against it needs a coordinated international response.^1,4,7^ The important note about this virus is its transmission to healthy people from asymptomatic persons. Moreover, some patients transmit it even after disease recovery.^1^ Due to the potential zoonotic nature of this virus and its capability for transmission by aerosols or droplets, SARS-CoV-2 has been considered a strict BSL3 pathogen for research purposes.^6^

 With attention to the importance of the subject, searching the neutralizing antibodies (Nabs) for CoV-2 is one method for treatment of this emerging disease. Antibodies could work by two mechanisms: Direct neutralization of target viral antigen and effective indirect mechanisms (e.g., complement-dependent cytotoxicity [CDC] and antibody-dependent cell-mediated cytotoxicity [ADCC]).^8^

 For the detection of various antigens, polyclonal antibodies (pAb) are widely used for diagnostics, therapeutics, and also research.^9^ Polyclonal sera (which include antibodies with different specificities for various immunogenic epitopes) are obtained from immunized animals such as rabbits, goats, and sheep. Depending on the immunized animal and immunization quality, produced antibodies may be different from each other.^10^ When the goal is detection the antigen with various epitopes, pAbs are preferred to monoclonal antibodies (mAbs). Purification of these antibodies is useful for many kinds of diagnostic methods. They could be used as ligands for immunoaffinity-column preparation in biological and biochemical research. They are used for coating or labeling reagents in immunoassay tests (e.g., Enzyme-linked immunosorbent assay[ELISA], immunoradiometric assay, western blotting, radioimmunoassays), and other applications, too.^11,12^ With this regard, many attempts are doing for developing antibody-based tests to detection of SARS-CoV-2 by companies and laboratories (e.g., GeneTex, Cellex, etc).^13^

 With attention to the mentioned subjects and due to the current situation caused by the distribution and mortality of SARS-CoV-2, the goal of this study was pAb production in response to concentrated antigen. Thereafter, the performance evaluation of produced antibody was performed by SDS-PAGE, ELISA, and western blot methods.

## Methods

###  Virus isolation from cell culture 

 Formalin inactivated (1:2000) SARS-CoV-2 virus strain (D614 mutant) with TCID50/mL ~6 and the cycle threshold (CT) value 12 in real-time PCR (ABI, USA) was prepared from a laboratory with the biosafety level 3.^12,14^

####  Virus enrichment by precipitation with polyethylene glycol (PEG)

 At this stage, we used the precipitation method by polyethylene glycol 6000 (PEG6000) (CinnaGen Co., Tehran, Iran) (as condensation polymer) together with NaCl (Merck, Darmstadt city, Germany) (as co-precipitant) for isolation and purification of viral particles from cell culture debris. To comparison the efficiency of various PEG/NaCl concentrations in virus purification, 20:4.4% (w/w), 22:4.6% (w/w), 25:5% (w/w) and 30:6.4% (w/w) mixture of these two compounds were used in different stages. Briefly, after centrifugation of cell culture at 5000 rpm for 5 minutes, the equal volume of the gathered supernatant was mixed with PEG/NaCl mixture. After incubation for 2 hours. on a shaker (IKA, South Korea) at room temperature, centrifugation was done at 13400 rpm for 20 minutes. The gathered pellet was dissolved in sterile PBS. Finally, all samples were analyzed by SDS-PAGE (Padideh Nogen Pars Co., Mashhad, Iran) electrophoresis in standard (non-reducing) conditions.^15-17^

####  Virus isolation using the sucrose cushion method

 Besides the previously mentioned method, the virus precipitation using a solution of sucrose cushion 6% was also performed for more virus purification and albumin elimination from the cell-culture medium. For this, the virus was initially precipitated by the best PEG/NaCl concentration (i.e., 30:6.4% w/w) and the following stages were performed:

1. A part of the gathered pellet was dissolved in sterile PBS. Then, for better antigen purification from cell culture, ammonium sulfate (Merck, Darmstadt city, Germany) (50% w/v) precipitation was performed. After centrifugation, the pellet was dissolved in 1 ml of sucrose cushion 6% solution. The sample was incubated at 37℃ for 30 minutes. Thereafter, centrifugation was done at 13400 rpm for 15 minutes. The gathered pellet was dissolved in 500μL PBS.
2. Another part of the pellet was dissolved in 1ml of sucrose cushion 6% solution (without any suspension in PBS buffer) and the following stages were performed as previous.^18,19^

The product of these two stages was also investigated using SDS-PAGE electrophoresis in standard conditions.

####  Determination the amount of the purified antigen 

 Protein concentration (whole viral antigen) was measured by the Bradford spectrophotometric method and bovine serum albumin (BSA) (SIGMA-ALDRICH, St. Louis, USA) was used as standard (Bradford reactant) in the range of 0-2.5 mg/mL^20^

 For visualization of the purified antigen bands, SDS-PAGE was done in non-reducing condition, as the previous stage (Padideh Nogen Pars Co., Mashhad, Iran). The resolving and stacking gel concentration was 11% and 4%, respectively. Electrophoresis was done in running buffer (192mM glycine, 25mM Tris-base, 1% SDS, pH 8.3; [CinnaGen Co., Tehran, Iran]) at 100 V for 90 minutes.^10,21,22^ Gel was stained by staining solution (1% Coomassie blue R-250, [Merck, Darmstadt city, Germany]) and de-stained by 7% acetic acid (Merck, Darmstadt city, Germany); 5% methanol (Merck, Darmstadt city, Germany); 88% water solution. The molecular mass standard (CinnaGen Co., Tehran, Iran) was run in parallel with other samples in order to calculate the molecular weights of the proteins.^21,23,24^

####  Antigen fractionation by ion-exchange chromatography

 The whole-viral antigen was fractionated by ion-exchange chromatography in NaCl gradients’ concentration in order to identify the most immunogenic fractions.^23^ For this, DEAE (diethyl amino ethyl)-cellulose (SIGMA-ALDRICH, St. Louis, USA) column was washed by Tris buffer (0.1M, pH 8.6) as column stabilizing buffer. Thereafter, the concentrated antigen was loaded onto the column. The elution of bounded components was performed by a two-fold NaCl gradient (0.0625-4M). Following, the protein concentration of the collected fractions was measured by Bradford spectrophotometric method as the previous stage.^23^

 Verification of the purified fractions was investigated using SDS-PAGE in reducing conditions, i.e., all samples were denatured by protein sample buffer containing 2-ME (7%) (Merck, Darmstadt city, Germany) before load onto gel.^11^

####  Enzyme-linked immunosorbent assay 

 Fractions’ specificity in the detection of anti-SARS-CoV-2 antibodies was determined via ELISA. Accordingly, all fractions were diluted in coating buffer for coating 1 μg of each fraction in ELISA microtiter plates (SPL life sciences, Gyeonggi-do, Korea, Republic), and incubated at 4℃, overnight. Thereafter, the wells were washed with BPST buffer (PBS plus tween 20). Blocking was performed with 4% skim milk (Merck, Darmstadt city, Germany) in PBS buffer at room temperature for 2 hours. Following, after 3 times washing of the wells with PBST, 100 μL of 1:50 and 1:10 dilution of positive serum samples from COVID-19 patients were added to each well, separately. Then, the plate was incubated at room temperature for 1 hour. and washed with PBST. HRP conjugated anti-Human antibody (100 μL) was added to each well in 1:2000 dilution. Following 1 hour. incubation at room temperature, reactions were developed by adding 75 μL of TMB (3, 3’, 5, 5’- tetramethyl benzidine) (IDvet, Grabels, France) as a substrate. Finally, the reactions were stopped by adding the sulfuric acid 2M (Merck, Darmstadt city, Germany). It should be noted that along with these samples, PBS buffer and negative serum samples were used as the negative control. An ELISA microtiter plate reader (AccuReader, Metertech Inc., Taipei, Taiwan) was used to determine the absorbance at 450 nm.

###  Injection of the purified antigen into rat

 In order to prepare the pAbs, the eight Wistar rats (6-7 weeks old) were used and fed regularly on a commercial diet.^11^ All stages of work, including immunization and blood collection, were performed according to the ethical principles for laboratory animals. Moreover, 2 rats were considered as a blank group and a mixture of sterile PBS (instead of antigen) with adjuvant was injected into them. An emulsion of 100 μg of antigen with Montanide ISA 70 adjuvant was used for the first immunization of test group and it was injected subcutaneously (s.c.) and intra-peritoneal (IP).^25,26^ Booster immunizations were performed subcutaneously by 50μg of antigen emulsified with Montanide ISA70 adjuvant.^26^ Overall, 4 injections were performed, and the interval of each booster was about 10 days.^20^

###  Blood collection and investigation of the antibody titer by ELISA

 By ending the injections, anesthesia was performed using xylazine (Alfasan, Woerden, the Netherlands): ketamine (BREMER Pharma, Warburg, Germany) (3:1 v/v) in the appropriate amount of sterile PBS, and the blood was collected from the tail of the immunized and blank rats. The collected blood samples (without any anti-coagulant agents) were placed at 4℃ for 12 hours. and was allowed to clotting in cold, for serum preparation. Then, centrifugation was performed at 5000 rpm for 10 minutes; the gathered serums were collected and stored at -20℃ until the assay time.^10,24,25^

 The titer of the anti-SARS-CoV-2 antibody was assayed by the indirect ELISA method as mentioned above. Based on the results of the previous stage, a mixture of the best fractions with good interactions with COVID-19 antibodies was used here. Thereafter, an ELISA microtiter plate was coated with 100μL of these fractions, then it was blocked using 0.3% Tween 20 (Merck, Darmstadt city, Germany) in PBS at 37℃. Diluted rat serums (1:100) were added to each well. Finally, 100 μL of HRP conjugated anti-mice antibody (1:2000 dilution) was added to each well.^25^ The subsequent steps were performed as previously, and the absorbance was measured at 450 nm. It should be noted that in addition to PBST, serum samples were treated with PBST + 2ME buffer for 1 hour. at room temperature before adding to wells to determine the antibody type.

 In addition to the evaluation of the produced pAbs performance via its interaction with the purified whole-cell viral antigen, we used commercial kits coated with recombinant spike protein (RBD) of SARS-CoV-2 (Cube Biotech co., Monheim, Germany). Other stages were similar to the study of purified viral antigens.

###  Primary purification of antibody by ammonium sulfate precipitation

 For purification of immunoglobulins from immunized rat serum and elimination of some impurities, the protein solution was precipitated using 33% (v/v) ammonium sulfate. For this, saturated ammonium sulfate solution was gently added to serum samples and incubated on ice for 30 minutes at 85 rpm. This solution was centrifuged for 20 minutes at 4℃. The supernatant was discarded and the pellet was dissolved in 40% (v/v) ammonium sulfate. After centrifugation, the enriched immunoglobulin pellet was dissolved in sterile PBS. This solution was subjected to dialysis against 1 L of phosphate buffer (0.02M) at 4℃, overnight.^27^

###  Final antibody purification using ion-exchange chromatography 

 Dialyzed solution was loaded onto a chromatography column packed with DEAE-cellulose. Samples were collected as 2 mL fractions in separate microtubes. These purified antibodies were subjected to non-reducing and reducing conditions of SDS-PAGE as mentioned above.^11,25,26^

###  Determining the produced antibody specificity against SARS-CoV-2 antigens via Western blotting 

 Western blotting is essentially being used for verification of protein antigenicity and specificity of the purified proteins. At first, the concentrated viral antigens by PEG/NaCl method were subjected to lysis using a lysis buffer containing 2ME. Following the protein separation of the lysed viral particle using SDS-PAGE (as in previous stages), the gel was immediately immersed in transfer buffer (13mM Glycine, 95mM Tris, and 20% methanol) without gel staining. Thereafter, the proteins were transferred to a PVDF membrane at 20 V for 2.5-3 hours (Paya Pajohesh Co., Tehran, Iran). Blocking was performed using 4% (w/v) skim milk in PBS buffer and it was incubated for 12 hours at 4℃. Following the washing with PBST, the gathered rat serums were added to the membrane and incubated at room temperature for 1 hour. Thereafter, washing was performed, and a diluted HRP-conjugated anti-mice antibody (1:2500) was added for antibody detection. Incubation was done at room temperature for 1 hour. The washing was repeated and the 4-chloro-1-naphthol (4CN) (Merck, Darmstadt city, Germany) solution was added as the enzyme substrate. After incubation at room temperature for 20 minutes in dark, washing was performed as the previous stages.^14,20,22,26,28-32^

###  Statistical analysis

 The gathered results of all stages (virus concentration, protein amount and ELISA of fractions, ELISA of the collected serums) were analyzed using SPSS software version 26.0, and their significance level was evaluated.

###  Theory

 In this study, our hypothesis was as follows:

- The performed method could produce a relatively pure antigen for immunization and induction of pAbs and it does not have any need for expensive methods and instruments.
- It was also hypothesized that this Ag has comparable results with the commercial antigens.
- Purified pAbs have good interaction with the specific antigens and do not have any cross-reaction.

## Results and Discussion

 As the seventh member of enveloped, positive-strand RNA viruses, the SARS-CoV-2 is capable to infect humans.^33^ The important subject about the SARS-CoV-2 is its faster distribution among doctors and family groups than the observed state of the SARS outbreak in 2003.^1^ So, this study was designed as follows. At first, the suitable method for purification of inactivated SARS-CoV-2 antigen was investigated for injection into rats. Thereafter, the efficiency of produced polyclonal antibody was evaluated.

###  Standard curve 

**Figure 1:**
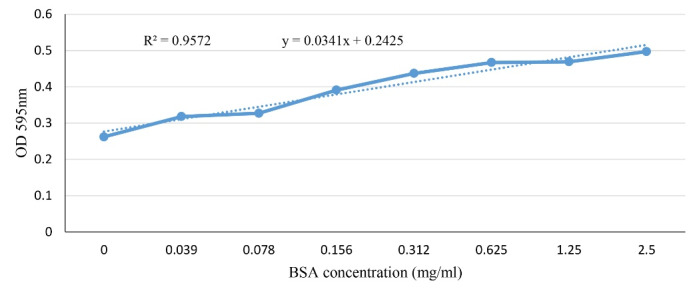
BSA Standard curve

 The BSA standard curve (in the range of 0-2.5 mg/mL) was shown in Figure 1. At all stages of this study (i.e., antigen precipitation, antigen fractionation, and antibody purification) the protein amount was calculated based on the calculated equation by this curve.

###  SDS-PAGE of precipitated antigens

 Preparation of purified viruses with high quantity and quality for structural, biochemical, virological, and biopharmaceutical studies needs efficient and simple downstream methods for the concentration and purification of viral particles. On the other hand, successful virus-like particles (VLP) preparation proposes a promising method for viral vaccine production. However, it is not possible to use the same purification approaches to many samples and each approach needs to be optimized.^34^ There are several parameters that should be considered for the selection of an appropriate method (e.g. cost, yield, product purity, and easy scaling up).^35,36^ Precipitation by various chemicals, along with the subsequent application of low-speed centrifugation, has been successful in promoting viral aggregation.^36^ Among these, PEG is a condensation polymer with several chemical properties which make it useful for various applications. PEG precipitation method is rapid, simple, and reproducible, it reduces the sample volume and it does not need expensive equipment (e.g., ultracentrifuge) or complex manipulations.^34^ Moreover, it has been found that the produced antigens are stable for many months at -70℃ in suspension or coated ELISA plates.^16^ Viral antigen precipitation is usually achieved by PEG in the presence of high concentrations of monovalent salt such as NaCl. Indeed, salts decrease the available water molecules for interaction with viruses and help to better aggregation and precipitation.^35^

**Figure 2:**
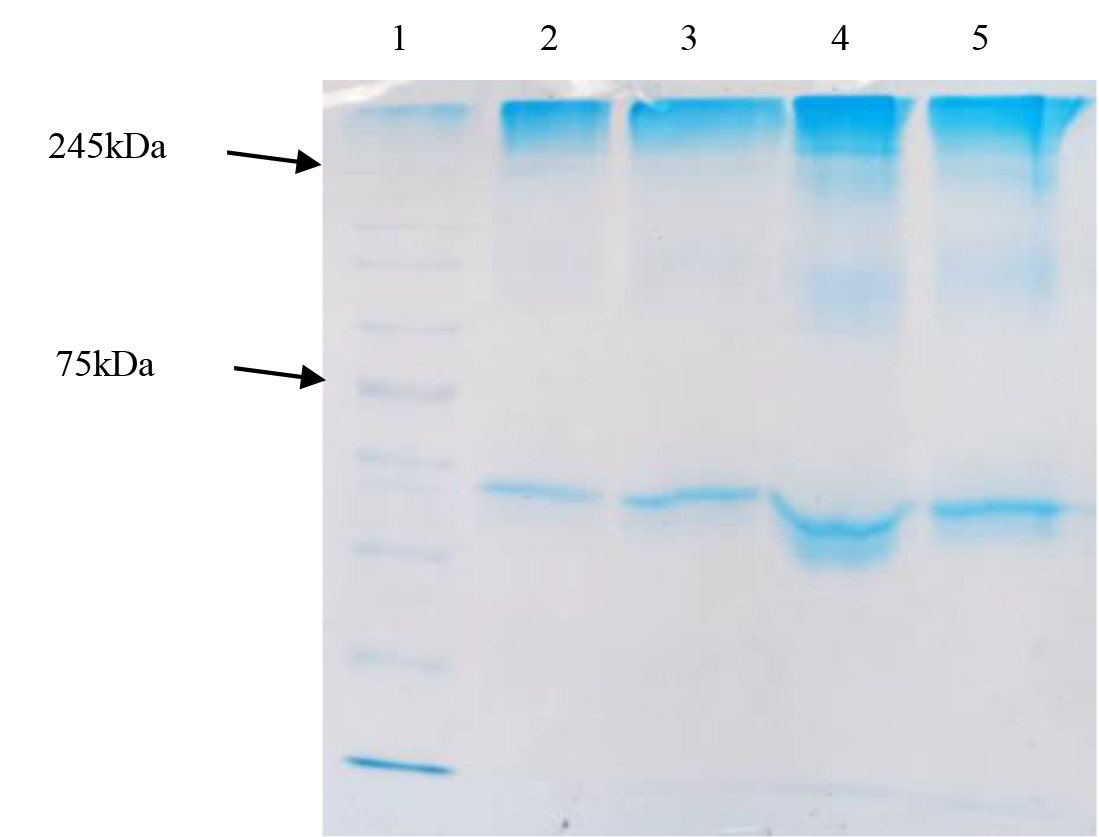
The result of SDS-PAGE of precipitated virus (in various PEG/NaCl concentrations) 1: Protein marker (10-250kDa); 2-5: Precipitated virus by 20:4.4%, 22:4.6%, 25:5.5% and 30:6.4% of PEG/NaCl, respectively.

 As mentioned previously, after enrichment by PEG/NaCl precipitation method, SDS-PAGE electrophoresis was performed for ensuring the antigen presence in inactivated viral cell culture. The gathered results showed the virus band in ~245 kDa as expected for SARS-CoV-2 protein. Moreover, it was shown that 30:6.4% of PEG/NaCl had the most efficiency for viral protein purification. SDS-PAGE also confirmed this result (Table 1 and Figure 2). However, the statistical analysis results did not show any significant difference between various PEG/NaCl concentrations (*P* > 0.05).

**Table 1:** The protein amount of concentrated virus (mg/mL)

**The various concentration of PEG/NaCl for virus concentration**	**The amount of extracted protein (mg/mL)**
20:4.4	2.19
22:4.6	2.99
25:5.5	4.83
30:6.4	7.85

 In the application of this method, Hagen et al^15^ used it for concentrating the hepatitis A virus for vaccine production in their pioneer study. The results showed that PEG concentrations above 4% (w/w), high salt concentrations (more than 0.75M), and temperatures ranging from 2-8℃ for 1 hour. were the best conditions in virus purification. In the current study, various PEG/NaCl concentrations showed a different efficiency in virus isolation from cell culture, too. The study of Simard et al^16^ also showed that produced antigen of the Canadian strain of caprine arthritis-encephalitis virus using PEG was suitable for ELISA with 100% specificity. Alexander et al^17^ also used a protocol for precipitation of SARS-CoV-2 stocks using PEG, without any requirement to ultracentrifugation. Application of this method caused a ~1.5log_10_ increase in SARS-CoV-2 titers in VeroE6 cells. This protocol has also been used for the isolation of SARS-CoV-2 with high efficiency from wastewater samples by some researchers such as Farkas et al^37^ and Dumke et al.^38^

 Unlike the effective use of PEG for simplifying the downstream purification stages, polymers may also be precipitated along with viral particles. To overcome such problems, several approaches are used in combination and in sequential order.^34,36^ For example, the direct pelletation of viral particles using ultracentrifugation on sucrose remains a common technique for concentrating enveloped viruses.^36^ However, the requirement to ultracentrifuge could be an instrumental barrier. In this regard, alternative methods have been reported that bypass ultracentrifugation.^39^ For example, Jiang et al^39^ succeed in concentrating lentivirus with high titer using sucrose gradient centrifugation at 10 000 g relative centrifugal force. This speed is reachable by ordinary centrifuges. Moreover, it was determined that the optimal sucrose concentration was 10%. Nevertheless, in some studies, such as Hutornojs et al,^36^ this method did not cause a significant viral concentration.

**Figure 3:**
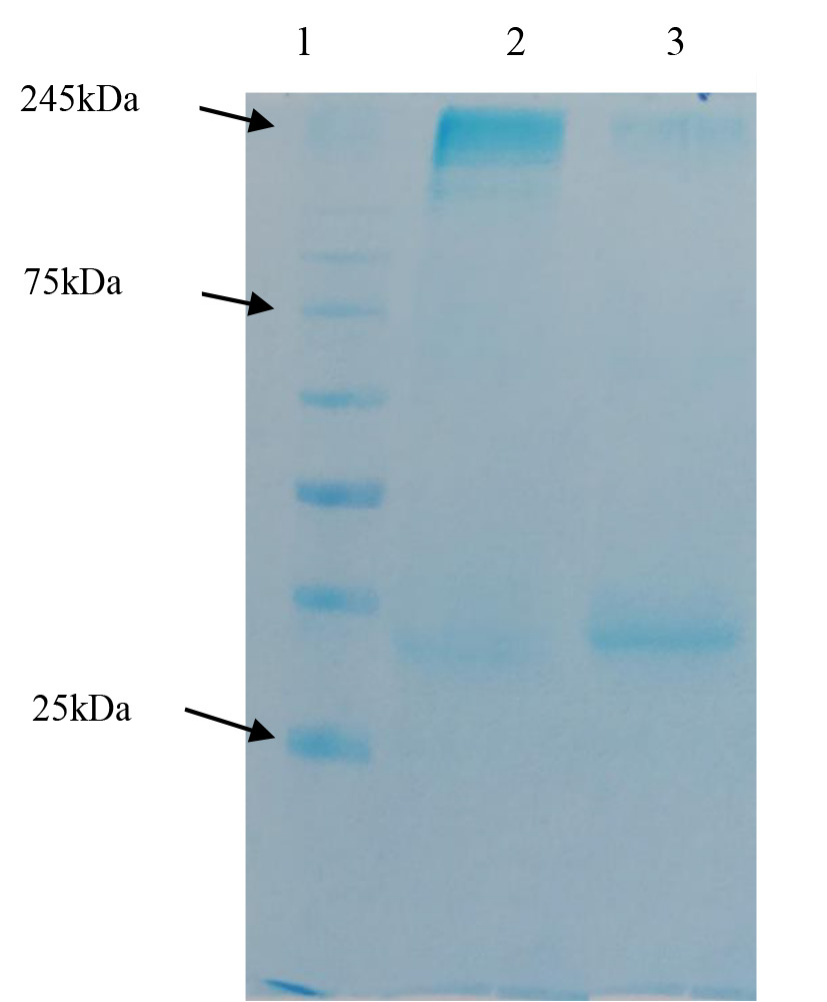
**1:** Protein marker (10-250kDa); **2: **Precipitated virus by sucrose 6%; **3:** Precipitated virus by ammonium sulfate and sucrose 6%

 As it was said, besides PEG/NaCl concentrating method, evaluating the precipitation method efficiency in albumin elimination from concentrated viral particles was also performed via two other experiments by sucrose cushion 6%, in our study. The results showed that this method caused the elimination of more impurities from concentrated virus without any requirement to ultracentrifugation, so a purer viral antigen could be attained by it (Figure 3). However, it should be noted that this amount needs to be optimized for practical applications.

###  Antigen fractionation 

 Several methods (such as ion-exchange chromatography) have been used for fractionation, purification, structural analysis, and detection of proteins.^23,27^ Introduction of a protein solution into a chromatography column compacted with ion-exchange resins makes electrostatic bonds between negative and positive charges of the proteins and resins, respectively.^40^ Efficient binding of proteins into the ion-exchange resins is dependent on various factors (such as the surface charge distribution, net charge, hydrophobicity of protein, van der Waals interactions, and selection of the sorbent materials) which are in turn affected by pH and the ionic strength of buffer.^27,41^ This partial solubility in pure water, various salt solutions, or ethanol may be used as a base fractionation technique.^27^ Accordingly, selective elution of ion-exchange resins is achieved by slowly increasing the ionic strength (to competitively disrupt ionic interactions between the column matrix and the proteins) or by pH change (to lose the charge of reactive groups on the protein surfaces).^42^ In this regard, this method has also been used for virus purification in our study.

 After fractionation by mentioned method, the absorbance of these fractions was measured. According to the results, different absorbances was observed among the various fractions. However, statistical analysis did not show any significant differences between various fractions (*P* > 0.05).

**Figure 4:**
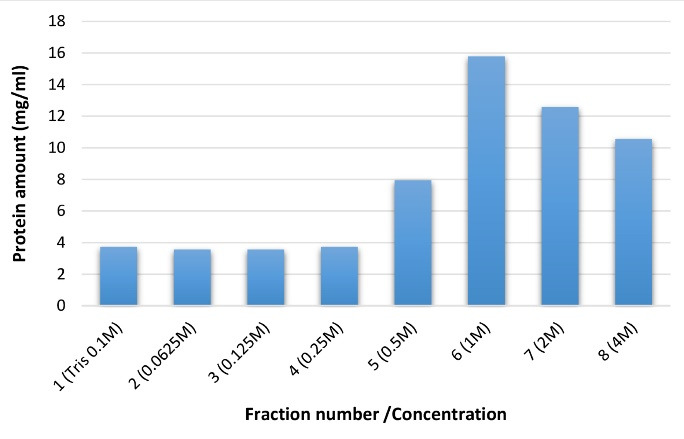
The protein amount of antigen fractions

 In the current study, the measurement of the protein amount of each fraction and its comparison with standard samples (BSA) showed that each fraction had a different protein amount following the column chromatography (Figure 4). This phenomenon could be explained by the fact that each fraction contained proteins with a different net charge. In fact, according to Figure 4, the releasing trend of viral fractions indicates that the first and second fractions have a positive charge; therefore, they are released at the first. On the other hand, the most protein amounts were attained in the sixth, seventh, and eighth fractions, which were probably eluted later due to their more negative surface charges.

**Figure 5:**
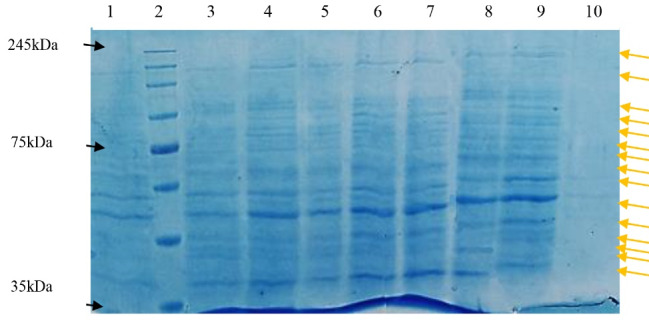
Fraction number 1 (Tris 0.1M); **2:** Protein marker (10-250kDa); **3:** Fraction number 2 (0.0625M); **4:** Fraction number 3 (0.125M); **5:** Fraction number 4 (0.25M); **6:** Fraction number 5 (0.5M); **7:** Fraction number 6 (1M); **8:** Fraction number 7 (2M); **9:** Fraction number 8 (4M); **10:** KCl-wash of column. Arrows shows the corresponded bands of each fraction.

 Moreover, the results showed that these fractions produced protein patterns containing discrete bands with molecular weight ranging from > 35 to < 245 kDa (Figure 5).

 Since it is possible that each antigen harbors various fragments with different immunogenicity features, Khosravi et al^23^ also purified fractions of scorpion venom using ion-exchange chromatography and eluted the bounded elements by a linear gradient of NaCl (in the range 0.1-0.2M). The results indicated that the released fraction by 1.25M NaCl had the most protein amount. Treatment of the chickens with venom fractions showed the maximum and minimum antibody titer in the fifth (0.75M) and seventh (1.25M) fractions, respectively.

 Oksanen et al^34^ also used DEAE column chromatography with a linear gradient of NaCl (0-1.5M) for purification of PRD1 bacteriophage. The results indicated that the most protein concentration and virus titer was gained at the NaCl concentration equal to 0.89M. These researchers stated that the utilization of ion-exchange chromatography in combination with some methods (e.g., PEG precipitation) is an effective and more rapid method in comparison to centrifuge-based approaches only (without any need for ultracentrifugation). On the other hand, separating empty and full viral particles using this approach by elution with NaCl gradient (10-300mM) was investigated in Dickerson et al^43^ and the best condition for isolating full viral particles (with ~94% efficiency) was determined at 9mM MgCl_2_, 50mM NaCl and pH 9.0.

 It should be noted that this method has not only been used for the purification of antigenic proteins but it has also been used for the purification of the carrageenase, alkaline phosphatase, and anti-fungal proteins (as biocontrol agents), and it was successful in separating the different protein fractions.^41,44,45^ The results of the current study also showed that different viral fractions were gathered by elution buffers with various ionic strengths. These fractions were different from each other based on their affinity to serum antibody and the protein amount.

 With reference to ELISA results and by comparison between the gathered data with the negative control, data analysis also showed that among 11 fractions, most of them were immunogen. However, the sixth (1M NaCl), the seventh (2M NaCl), and the eighth (4M NaCl) fractions have the most ability to interact with antibodies; accordingly, a mixture of these fractions was used to assay the efficiency of pAbs. But no significant difference was shown between them (*P* > 0.05).

 Moreover, it should be noted that antigen fractions resulted in more OD in 1:10 dilution in comparison to 1:50 dilution (Table 2). The significance of this difference was also confirmed by statistical analysis (*P* < 0.05).

**Table 2:** The ELISA results of antigen fractions with positive human serums.

	**1**	**2**
A	0.109	2.809
B	0.078	2.988
C	0.064	2.054
D	0.07	2.786
E	0.075	2.864
F	0.118	3.001
G	0.112	3.033
H	0.112	3.061
I	0.056	0.057

The A-H rows indicate the reaction of 1-8 fractions with: 1:50 dilution (Column 1), and 1:10 dilution (Column 2) of human serums; I row indicates the control (-) reaction.

###  Polyclonal antibody preparation 

 After the first introduction of antibody-based therapy, many researchers attempted to use immunoglobulins in the detection and treatment of various diseases.^11^ In the case of laboratory immune assays, an assay scheme with pAbs has certain benefits than those which are dependent on the detection of an epitope by monoclonal antibodies (mAbs).^34^ In this regard, in Ascoli^46^ the B.1.1.7 strain of SARS-CoV-2 (which harbors the 17 mutations) was detected by pAbs, while no of these mutations did not inhibit pAbs ability to detect SARS-CoV-2 N protein.^12^

 Commonly, the antibody titer will increase after each immunization. In the current study, injection of the emulsified antigen with Montanide ISA70 adjuvant was performed to increase the stability and prolong antigen release. The antibody titer was high after the fourth injection. In this regard, the results of Mehrazin et al^20^ showed that after the third injection in rats, the antibody titer against *Bacillus anthracis*’s PA antigen was equal to the fourth booster in the rabbit. It could be concluded that the significant antibody titer could be consequently reached in a lower time by rats than the rabbits. Alizadeh et al^10^ obtained the same results for specific antibodies against *Mycobacterium* antigen in the New Zealand rabbit, too.

 The results showed that rats produced a good response to the injected antigen after the third booster with the purified antigen. With reference to Table 3 and with a slight difference between two treatments (PBST with and without 2ME), it could be said that produced antibodies are IgG. Statistical analysis also confirmed these results (*P* > 0.05). These results indicate a strong antibody response to antigens which developed after about 12 days from the last booster. Therefore, this assumption is reasonable that this response was due to enough affinity maturation and antibody specificity against immunogen.

**Table 3:** The ELISA results of produced polyclonal antibodies with the purified antigen after the third boost

	**1**	**2**
A	0.296	0.288
B	0.206	0.208
C	0.245	0.296
D	0.247	0.246
E	0.256	0.236
F	0.107	0.095
G	0.093	0.085
H	0.059	0.078

Columns 1 and 2: Serum samples treated with PBST + 2ME and PBST, respectively; F and G: Serum samples of the blank group. H: control -.

 The interaction results of produced antibody with recombinant spike antigen (Table 4) showed a strong response using its main concentration (2.861) and different dilutions (column 3). These results indicate that this antibody could also act against recombinant protein and therefore has the potential to use in related diagnostic and productive works.

**Table 4:** The ELISA results of produced polyclonal antibodies with the recombinant spike protein

	**1**	**2**	**3**
A	0.023	0.049	0.973
B	0.018	0.034	0.806
C	0.021	0.025	0.565
D	0.014	0.021	0.433
E	0.019	0.028	0.364
F	0.011	0.014	0.233
G	-	2.861	-

Columns 1 and 2: control -; 3: Serums from immunized rats with different dilutions (A: 1:20 to F: 1:640). G: control + (immunized rat serum)

###  SDS-PAGE of the produced antibody

 There is a long history of research that consider various approaches to reach an excellent method for isolation and purification of antibodies.^11^ Among these, precipitation method is an effective approach for protein concentration based on their sensitivity to the ionic strength, different pI, and other features.^42^ For example, Liu et al^22^ purified the produced anti-HAb18G/CD147 pAbs in rabbits using the ammonium sulfate precipitation and antigen-immunoaffinity chromatography methods. These purified antibodies showed a good performance in sandwich ELISA. Similar to the current study, these authors also used indirect ELISA with 1:100 dilution of antigen.

**Figure 6:**
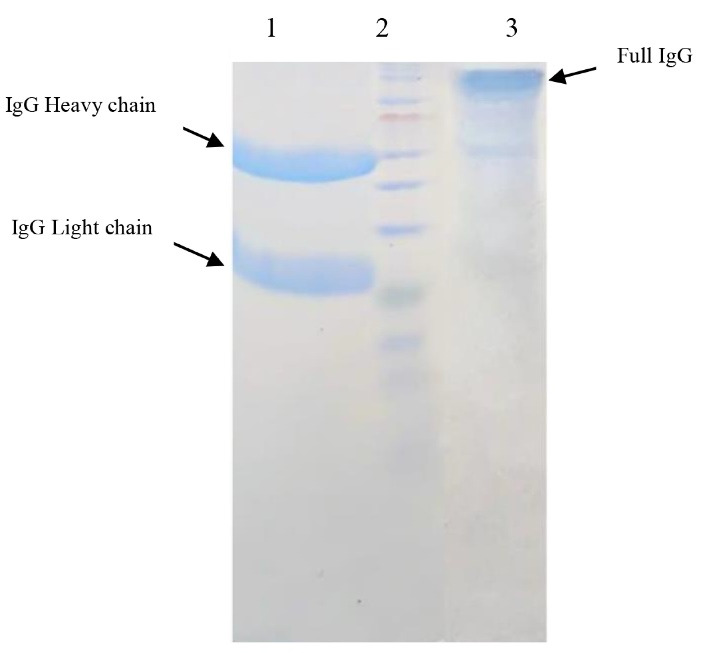
The SDS-PAGE results of purified antibody. **1:** The purified pAb treated with 2ME; **2:** Protein marker (10-250kDa); **3:** The purified pAb without 2ME

 After purification by column chromatography, the produced rat antibodies were subjected to SDS-PAGE in non-reducing and reducing conditions. In non-reducing conditions, this antibody showed a single band with an estimated molecular mass of ~150kDa which is corresponded to the rat IgG (Figure 6). Similar to the antibodies from other mammalians, the purified rat IgG had two chains and produced two bands with an estimated molecular mass of 50 kDa (for heavy chain) and 25kDa (for light chain) in the presence of a reducing agent (in this study, 2-mercapto ethanol) (Figure 6). According to the standard equation, the amount of produced antibody was calculated as 12.16 mg/mL.

 ELISA has also been used in various studies to investigate the ability of antigen detection by designed antibodies.^28^ To investigate the specificity of anti-SARS-CoV-2 RBD/spike scFv antibodies, this method was used by Parray et al.^8^ The specificity and functional activity of the purified II62-scFv were determined via ELISA and it did not show any interaction with unrelated proteins such as BSA and envelope proteins of Chikungunya and HIV. Ho et al^47^ also used western blotting and ELISA methods to analyze the antigenicity of recombinant S-protein against the collected serums from SARS patients or Spike-immunized rabbits. This approach was also adapted by Jegouic et al^48^ for evaluating the performance of the produced antibodies against SARS-CoV-2 using coated ELISA microtiter plates with expressed S1 antigen in *E. coli* and baculovirus system. They reported that this method caused the identification of 14% of cases as positive and 77% as negative. In the current study, it was found that the produced pAbs were able to detect the recombinant spike protein, whole-viral antigen, and the corresponding fractions in ELISA and western blotting tests.

###  Western blot

**Figure 7:**
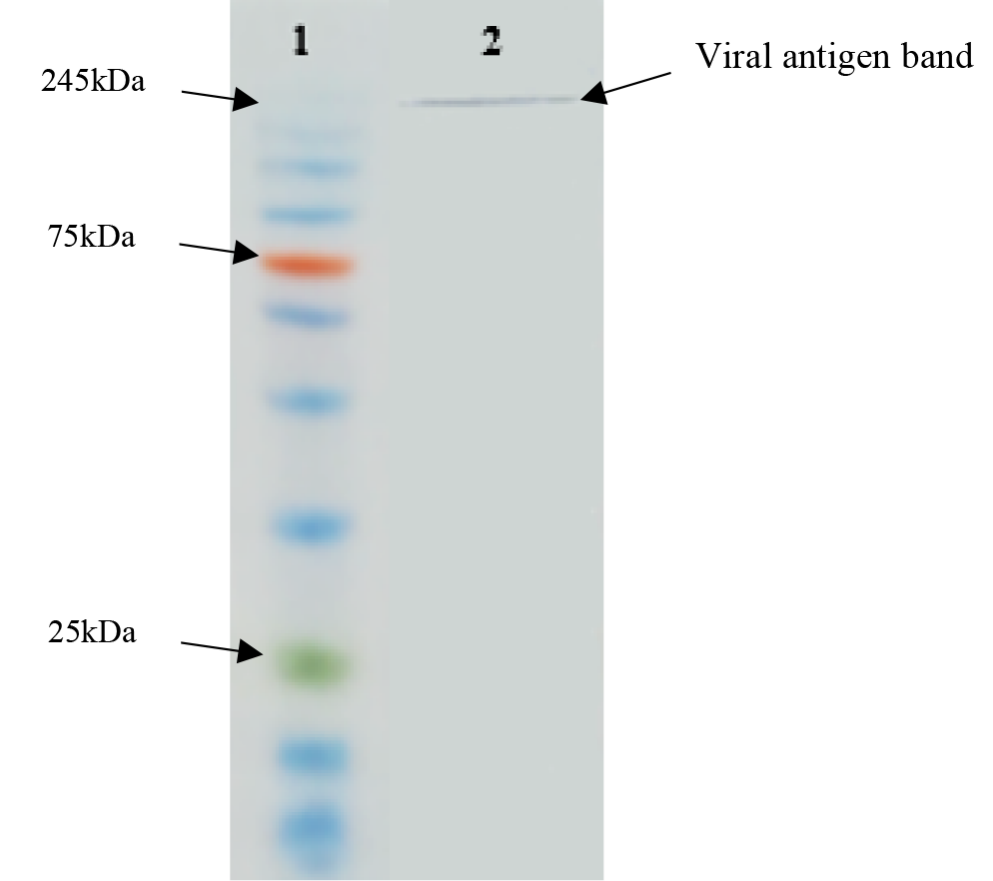
** 1: **Protein marker (10-250kDa); **2: **The interaction between Ab-lysed viral particle

 The specificity of the purified pAbs was verified using western blotting. The obtained results showed that the purified pAbs were able to detect the antigen band with an estimated molecular mass of 245 kDa (Figure 7).

## Conclusion

 Unlike mAbs introduction, the application of pAbs in the diagnostic tests (e.g., ELISA, western blot, and flow cytometry) has many usages and they are on the selling list of many companies.^49^ The current study showed that sucrose gradient could yield higher purity of viral antigen and it could be a comparable method with sophisticated and expensive methods (e.g., ultracentrifugation). In this study, high titers of anti-SARS-CoV-2 serum were attained, and these antibodies were purified with ammonium sulfate and antigen-affinity chromatography in the following stages. Moreover, it was found that this antibody has the ability to suitable reaction with the recombinant spike protein in commercial kits. Therefore, it has the potential to use in related diagnostic and productive works, too. In the following phase, this study will focus on the preparation of the recombinant monoclonal antibodies (mAbs) against the specific antigen of SARS-CoV-2 and we hope that it could be used in the treatment (as an alternative to plasma therapy) and detection of the infected cases with this emergent virus.

## Competing Interests

 The authors declare there are no conflicts of interest.

## Ethical Approval

 This work was performed at the Shahid Chamran University of Ahvaz and the Ethical Committee of this institution has approved the work (Ethical code: EE/1400.3.02.25165/Scu.ac.ir).
